# Broadening horizons: the role of ferroptosis in polycystic ovary syndrome

**DOI:** 10.3389/fendo.2024.1390013

**Published:** 2024-08-02

**Authors:** Min Wang, Bo-Qi Zhang, Shuai Ma, Ying Xu, Dong-Hai Zhao, Jing-Shun Zhang, Chun-Jin Li, Xu Zhou, Lian-Wen Zheng

**Affiliations:** ^1^ Department of Obstetrics and Gynecology, The Second Hospital of Jilin University, Changchun, China; ^2^ College of Animal Sciences, Jilin University, Changchun, China; ^3^ Department of Pathology, Jilin Medical College, Jilin, China

**Keywords:** ferroptosis, polycystic ovary syndrome, oxidative stress, metabolic disorders, expression, biomarkers

## Abstract

Polycystic ovarian syndrome (PCOS) is a common heterogeneous reproductive endocrine metabolic disorder in women of reproductive age characterized by clinical and biochemical hyperandrogenemia, ovulation disorders, and polycystic ovarian morphology. Ferroptosis is a novel type of cell death driven by iron accumulation and lipid peroxidation. Ferroptosis plays a role in maintaining redox balance, iron metabolism, lipid metabolism, amino acid metabolism, mitochondrial activity, and many other signaling pathways linked to diseases. Iron overload is closely related to insulin resistance, decreased glucose tolerance, and the occurrence of diabetes mellitus. There is limited research on the role of ferroptosis in PCOS. Patients with PCOS have elevated levels of ferritin and increased reactive oxygen species in ovarian GCs. Studying ferroptosis in PCOS patients is highly important for achieving personalized treatment. This article reviews the progress of research on ferroptosis in PCOS, introduces the potential connections between iron metabolism abnormalities and oxidative stress-mediated PCOS, and provides a theoretical basis for diagnosing and treating PCOS.

## 1 Introduction

**Figure 1:**
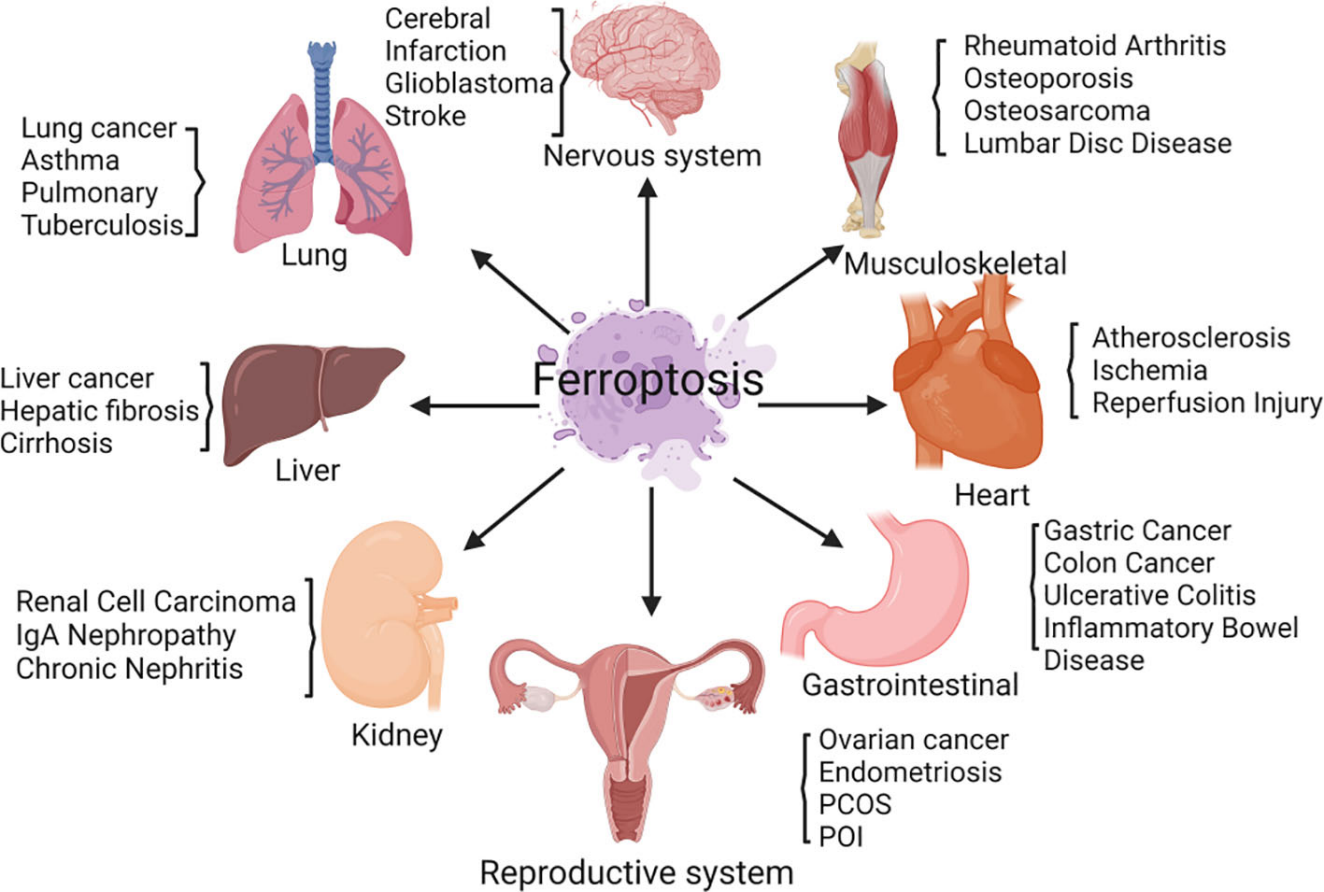
Ferroptosis is involved in regulating the occurrence and progression of various diseases, including those of the nervous system, respiratory system, cardiovascular system, diabetes mellitus, urinary system, and female reproductive system.

PCOS is a complex endocrine and metabolic disorder (1). The primary characteristics of PCOS patients are long-term anovulation or infrequent ovulation, insulin resistance (IR), hyperandrogenemia, polycystic ovarian morphology, and decreased female fertility (2–4). In addition to having abnormal reproductive function, many PCOS patients suffer from metabolic syndrome, IR, impaired glucose tolerance, obesity, atherosclerosis, and other metabolic abnormalities (5). At present, it is believed that the occurrence of PCOS is mainly related to genetic factors, environmental factors, and metabolic factors (6, 7). The specific causes of PCOS have not been fully elucidated (8, 9). Ferroptosis is caused by iron buildup and lipid peroxidation. It differs from cell apoptosis and necrosis (10). Ferroptosis is involved in regulating the occurrence and progression of diseases such as cancer, respiratory system diseases, cardiovascular system diseases, diabetes mellitus, and urinary system diseases and plays a key role in disease treatment (11) (**Figure 1**). There is limited and incomplete research on ferroptosis in the reproductive system. The role of ferroptosis in the reproductive system deserves further investigation (12, 13). Ferroptosis in PCOS is accompanied by the dysregulation of mitochondrial dynamics, the promotion of an inflammatory response, and the intensification of oxidative stress (14–16). This article reviews the correlation between ferroptosis and PCOS, providing ideas for exploring the underlying mechanisms of PCOS.

## 2 Ferroptosis

In 2012, Dixon et al. proposed ferroptosis as a novel iron-dependent programmed cell death mechanism that is distinct from apoptosis, necrosis, and autophagy (17) (**Table 1**). Its main characteristics are as follows (1): During the process of cell death, a large amount of iron accumulates, and lipid peroxidation occurs, activating signaling pathways such as oxidative stress (18) (2); In the ultrastructure, cell atrophy, membrane rupture, and mitochondrial membrane wrinkling, and no significant nuclear morphological changes are observed (19). Ferroptosis is a nonenzymatic and enzymatic reaction that occurs under iron catalysis, leading to lipid peroxidation of cell membranes (20). Polyunsaturated fatty acids (PUFAs) are the main targets of lipid peroxidation of cell membranes (21). Glutathione peroxidase 4 (GPX4) regulates ferroptosis (22). Under antioxidant conditions, GPX4 can reduce the accumulation of intracellular reactive oxygen species (ROS) and reduce the sensitivity of cells to ferroptosis, thus affecting the occurrence of ferroptosis (23).

**Table 1:** Comparison of cell death pathways.

Cell death pathway	Morphological changes	Biochemical characteristics
Ferroptosis	Rupture of the outer mitochondrial membrane, reduction or loss of mitochondrial ridges, and mitochondrial membrane density Increased, normal nucleus.	Iron ion and lipids Peroxide, ROS accumulation, system Xc- activation, GSH consumption.
Cuprotosis	The outer membrane of mitochondria is normal, with narrow and twisted cristae; Mitochondrial concentration, separation of inner and outer membranes, and increased matrix density; Mitochondrial envelope	Excessive intercellular copper and TCA cycle disorders
Pyroptosis	The main form of inflammatory necrotic cell death is membrane pore formation. Organelle loss, cell membrane rupture, release radioactive pro-inflammatory cytokines	Activation of caspase and gasdemin, release of neutrophil elastase and myeloperoxidase by alarge number of proinflammatory factors, activation of PAD4.
Necrosis	Plasma membrane rupture, cytoplasmic organelle swelling, chromatin concentration.	ATP depletion, protein hydrolysis and DAMP release involving calpain and cathepsin.
Apoptosis	Agglutination of chromatin, formation of apoptotic bodies, disintegration of cytoskeleton, reduction of cell and nuclear volume.	Caspase activation, PS exposure, mitochondrial membrane potential.
Autophagy	Damage or dysfunctional organelles and macromolecules are cleared from cells through autophagosome fusion, and enzymatic digestion. Formation of double membrane autolusosomes, including large autophagy, microautophagy and partner mediated autophagy	LC3-I to LC3-II conversion and substrate degradation.

ROS, reactive oxygen species; GSH, glutathione; DNA, deoxyribonucleic acid; PAD4, peptidylarginine deiminase 4; ATP, adenosine triphosphate; DAMP, damage-associated molecular pattern; PS, phosphatidyl serine; LC3, microtubule-associated protein light chain 3.

**Figure 2:**
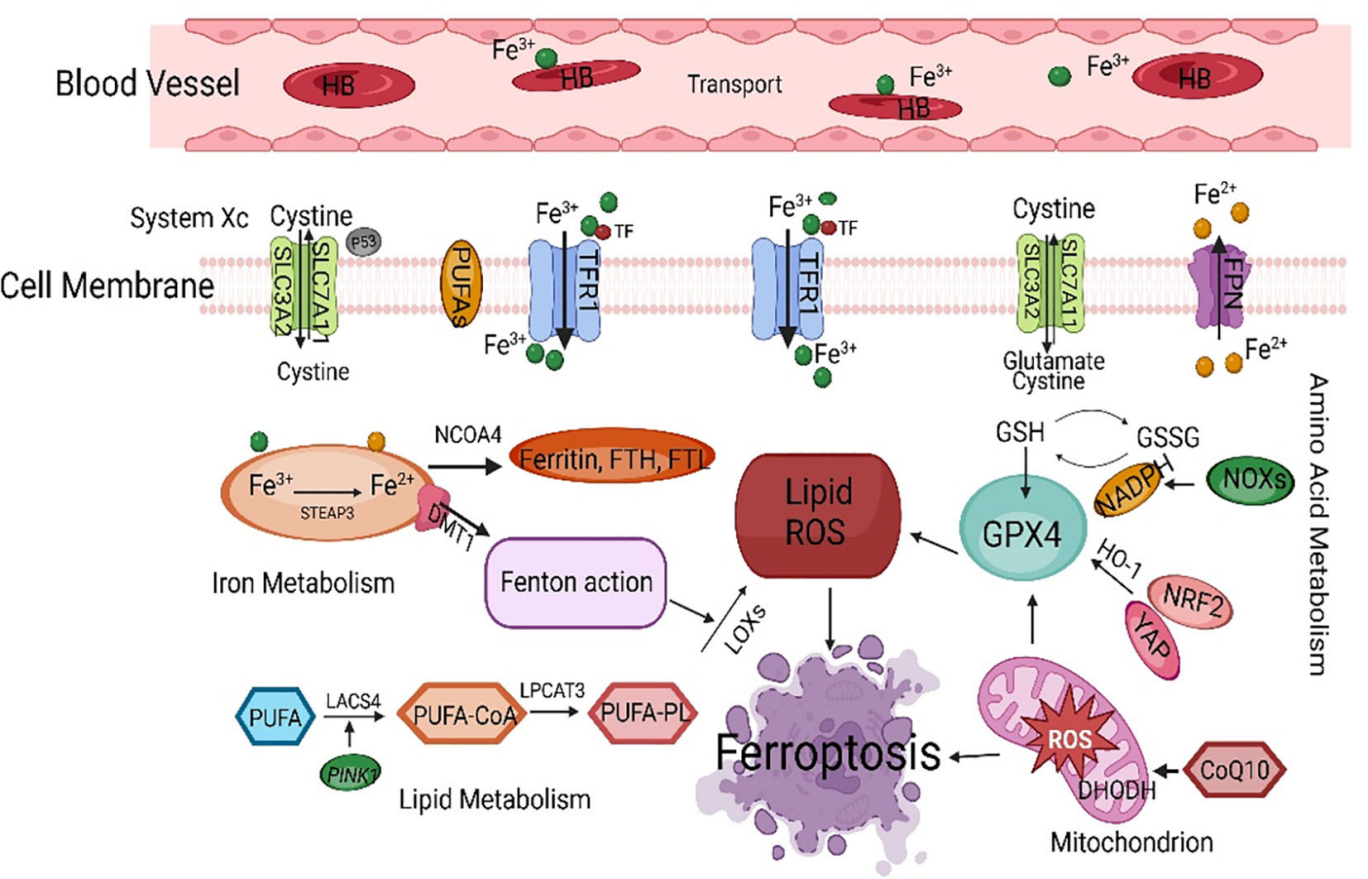
The main regulatory mechanisms of ferroptosis. SLC7A11, solute carrier family 7 member 11; PUFAs, polyunsaturated fatty acids; TFCR1, transferrin receptor 1; FPN, ferroportin; NCOA4, nuclear receptor coactivator 4; FTH, ferritin heavy chain; FTL, ferritin light chain; GSH, glutathione; GPX4, glutathione peroxidase 4; HO-1, heme oxygenase-1; NRF2, nuclear factor-erythroid 2-related factor 2; ROS, reactive oxygen species; LACS4, acyl-CoA synthetase long-chain family member 4.

The Fenton reaction first removes a hydrogen atom from poly-unsaturated fatty acid-phosphatidyl ethanolamine (24). This leads to the formation of carbon-centered phospholipids, which then react with molecular oxygen to generate PLOO·, which can remove hydrogen from another PUFA and form phospholipid hydroperoxides (25). If antioxidants such as GPX4 are unable to promptly convert phospholipid hydroperoxides to the appropriate alcohols, phospholipid hydroperoxides and lipid free radicals will react with polyunsaturated fatty acid-phosphatidyl ethanolamine to further promote the production of phospholipid hydroperoxides (26). Ultimately, this leads to the production of many secondary products, such as lipid peroxidation products, leading to ferroptosis (27). Ferroptosis mainly causes oxidative metabolic disorders of cell membrane phospholipids through iron metabolism, lipid metabolism, and amino acid metabolism (28) (**Figure 2**).

### 2.1 Ferroptosis and iron metabolism

#### 2.1.1 Iron uptake, storage, and release

Ferroptosis requires the involvement of iron (29). The sensitivity of cells to ferroptosis depends on the input, output, storage, and transport of iron. There are two forms of iron in cells: Fe^2+^ and Fe^3+^ (30). A large amount of ROS is generated during the mutual conversion of Fe^3+^ and Fe^2+^ through redox reactions, leading to tissue damage and ferroptosis (31). Fe^3+^ has strong stability and is responsible for the storage and transportation of iron in the body; Fe^2+^ has an electron transport ability, and proteins containing Fe^2+^ can participate in various redox reactions (32). Transferrin (TF) carries Fe^3+^ in the bloodstream and transfers iron in cells through transferrin receptor 1 (TFCR1). Fe^3+^ is reduced to Fe^2+^ by the six-transmembrane epithelial antigen of prostate 3, which is stored in the ferritin heavy chain (FTH) and ferritin light chain (FTL) (33). Fe^2+^ is stored in ferritin (Fn) and becomes a part of the labile iron pool, which plays a dominant role in ferroptosis. TFCR1 is considered a marker protein for ferroptosis, and knocking out TFCR1 can block ferroptosis. In the absence of enough iron in cells, an “iron starvation response” begins, increasing iron availability by increasing TFCR1 and decreasing the levels of FTH and ferroportin (FPN) (34).

#### 2.1.2 Iron homeostasis

Under normal circumstances, intracellular iron maintains a subtle balance through the iron transport system (35). Some iron is used for the biosynthesis of hemoglobin and iron sulfur clusters and for DNA synthesis. Cellular iron homeostasis also depends on iron regulatory proteins and iron-responsive elements (36). Iron regulatory proteins can bind to iron-responsive element mRNAs, regulating their translation process. Aberrant expression or dysfunction of the divalent metal transporter 1, TFRC1, and Fn genes and FPN leads to intracellular iron imbalance (37). Iron regulatory protein 2 can enhance the sensitivity of cells to erastin-induced ferroptosis by inhibiting the expression of FTH and FTL (38). When the plasma iron level meets the systemic iron demand, the liver will increase the secretion of hepcidin into the blood, reducing the plasma iron level and maintaining iron homeostasis in the body (39). Although every cell requires iron to generate energy, high iron levels can induce inflammation, oxidative stress, and lipid peroxidation in the cell membrane, leading to ferroptosis (40).

Iron homeostasis dysregulation is involved in ferroptosis, and multiple iron metabolism regulatory factors work together to maintain iron homeostasis (41). As mentioned, Fn autophagy is crucial for regulating ferroptosis (42). Nuclear receptor coactivator 4 (NCOA4) helps lysosomes breakdown intracellular Fn through autophagy, which releases free iron and causes oxidative damage (43). This selective autophagy process is called iron autophagy. Knockout of NCOA4 can inhibit ferroptosis caused by decreased amino acid or cysteine levels (44). Research has shown that knocking out autophagy-related genes 5 (ATG5) and ATG7 can reduce intracellular Fe^2+^ levels and lipid peroxidation, inhibiting ferroptosis (45). In addition to selective autophagy, activating the ubiquitin−proteasome system can promote Fn degradation. As an antioxidant, ubiquitin captures lipid peroxidation free radicals, prevents lipid peroxidation generation, and inhibits ferroptosis. Tang et al. reported that in treated rat hearts, ubiquitin-specific protease 7 increases iron uptake and promotes ferroptosis by activating the P53/TFRC1 pathway (46).

### 2.2 Ferroptosis and lipid metabolism

The conversion of lipids into membrane phospholipids and the occurrence of peroxidation are necessary conditions for ferroptosis (47). Lipid metabolism is closely related to ferroptosis. Most ROS related to ferroptosis originates from the Fenton and Haber-Weiss reactions, which then interact with PUFAs on the lipid membrane to form ROS (48). Phosphatidylethanolamine and arachidonic acid are essential membrane phospholipids that cause ferroptosis in the cell membrane. Lipoxygenases are nonheme iron-dependent dioxygenases that target polyunsaturated fatty acids (PUFAs). With the participation of iron in the cytoplasm, lipid free radicals are formed, which can directly oxidize PUFAs and PUFA-containing lipids in the biofilm, leading to cell damage (49). A decrease in lipoxygenase expression can also effectively improve ferroptosis induced by erastin (50). However, lipoxygenases cannot prevent ferroptosis induced by RSL3 (51). Although lipoxygenases do not have extensive regulatory effects on ferroptosis, they play essential roles in specific ferroptosis mechanisms.

Acyl-CoA synthetase long chain family member 4 (ACSL4) and lysophosphatidylcholine acyltransferase 3 are key enzymes involved in the process of lipid peroxidation (52). ACSL4 and lysophosphatidylcholine acyltransferase 3, which are involved in the synthesis of PUFAs, play essential roles in the ferroptosis pathway (53). ACSL4 binds PUFA to coenzyme A through acylation and further undergoes an esterification reaction with phosphatidylethanolamine under the action of LPCAT to generate PUFA-PE (54). Continuous oxidation reactions and consumption of PUFAs may alter the fluid structure of cell membranes, thereby increasing membrane permeability and ultimately leading to cell death. Inhibiting the expression of ACSL4 can increase the resistance of cells to ferroptosis and can be a target for inhibiting ferroptosis, which may provide new ideas for diagnosing and treating PCOS. Ferroptosis may play a role not only in pathological conditions but also in physiological processes. The normal development of the human fetal immune system depends on sufficient dietary intake of PUFAs (55). To summarize, studying the correlation between lipid metabolism and ferroptosis has essential research value (56).

### 2.3 Ferroptosis and amino acid metabolism

Ferroptosis is closely related to amino acid metabolism (57). The consumption of glutathione (GSH) can lead to the inactivation of GPX4 (58). Cysteine synthesizes GSH in the body through the cystine/glutamic acid reverse transporter (System Xc -) and the transsulfuration pathway (59). System Xc -, located on the cell membrane, is a heterodimer linked by disulfide bonds and contains the heavy chain subunits solute carrier family 3 member 2 and solute carrier family 7 member 11 (SLC7A11) (60). System Xc - is responsible for transporting extracellular cysteine into the cell. Glutamate exchanges cysteine at a 1:1 ratio, transporting intracellular glutamate to the outside of the cell (61). Cysteine provides the raw material for intracellular GSH synthesis (62). Another source of cysteine is cysteine sulfide, which is formed through the reverse sulfurization of methionine (63).

#### 2.3.1 Negative regulatory factors of System Xc -

Inhibiting cysteine uptake by System Xc - can inhibit the synthesis of GSH, leading to the accumulation of peroxides in the body and the induction of ferroptosis (64). System Xc—inhibitors such as erastin, sulfasalazine, sorafenib, and glutamate—are considered class I ferroptosis inducers, and inhibition of System Xc - leads to compensatory transcriptional upregulation of SLC7A11 (65). SLC7A11, an essential component of System Xc -, is an upstream regulatory factor of ferroptosis (66). SLC7A11 can reduce lipid peroxidation accumulation and prevent cells from entering the ferroptosis program by introducing cysteine and promoting GSH synthesis (67). Song et al. reported that Beclin 1 directly stops System Xc - activity by attaching to the core region of SLC7A11 and encouraging ferroptosis (68). Nuclear factor erythroid 2-related factor 2 (NRF2) is a crucial antioxidant transcription factor. Silencing NRF2 can significantly reduce the expression of SLC7A11 and heme oxygenase-1 (HO-1) and inhibit ferroptosis (69).

#### 2.3.2 GPX4: a core regulator of ferroptosis

Ferroptosis is related to the inactivation of GPX4 (70). As an important antioxidant in cells, GPX4 is a key ferroptosis regulator. GSH is used as a reducing substrate to promote the transformation of lipid peroxidation products into hydroxyl compounds, preventing ferroptosis in cells and protecting the structure and function of cell membranes from interference and damage (71). Under normal circumstances, fatty acid hydroperoxides can be converted into fatty acid alcohols through GPX4 mediation (72). Inhibiting GPX4 can interfere with intracellular iron homeostasis and reduce lipid peroxidation. The ferroptosis inducers RSL3 and erastin can both inactivate GPX4. RSL-3 blocks GPX4, which increases the levels of ROS and malondialdehyde (MDA) inside cells and promotes ferroptosis by blocking the SLC7A11/GSH/GPX4 pathway (73). Erastin indirectly inhibits GPX4 by inhibiting cysteine input, leading to cell membrane damage and death. Selenocysteine is an amino acid found in the active center of GPX4 (74). It can maintain GPX4 activity and help eliminate lipid peroxidation, which stops ferroptosis. Therefore, selenium deficiency can inhibit GPX4 activity and induce ferroptosis. GPX4 can also be turned off directly by squalene synthase, HMG CoA reductase, and other enzymes, which can change reduction reactions (75).

### 2.4 Ferroptosis and antioxidant pathways

Under physiological conditions, when cells undergo lipid peroxidation, multiple antioxidant pathways counteract this change. Research has shown that the intracellular ferroptosis antioxidant system is related to the SLC7A11/GSH/GPX4 signaling pathway (76). GPX4 reacts with GSH and lipid peroxidation products, efficiently clearing accumulated lipid peroxidation products and maintaining normal physiological functions. Ferroptosis suppressor protein 1 (FSP1) is a newly discovered inhibitory protein that inhibits GPX4 deficiency (77). The NAD(P)H/CoQ10/FSP1 signaling pathway is a crucial ferroptosis regulatory pathway (78). GTP cyclohydrolase 1 is involved in ferroptosis resistance, and its mechanism involves the generation of tetrahydrobipterin (BH4), which has redox activity, from GTP (79). BH4, a powerful antioxidant that captures free radicals, can promote CoQH2 regeneration and combat lipid peroxidation by activating downstream molecules such as GTP cyclohydrolase 1. These results indicate that the NADH-FSP1 CoQ10 and GTP cyclohydrolase 1/BH4 pathways work together with the SLC7A11/GPX4/GSH pathway to inhibit ferroptosis (80). Mao et al. reported that there is a ferroptosis system in mitochondria mainly composed of dihydrolactate dehydrogenase, which also induces lipid peroxidation in the mitochondrial membrane by promoting the reduction of CoQ10 to combat ferroptosis (81).

### 2.5 Ferroptosis and other regulatory mechanisms

#### 2.5.1 p53-mediated ferroptosis

Research has shown that p53 has a complex dual mechanism for regulating cellular ferroptosis through transcription and translation (82). P53 can inhibit cysteine uptake by downregulating SLC7A11, reducing GPX activity and GSH synthesis and subsequently causing intracellular ROS accumulation and ferroptosis (83). On the other hand, p53 can promote the activity of glutaminase 2 and spermidine/spermine N1-acetyltransferase 1 (SAT1). Glutaminase 2 catalyzes the degradation of glutamine to glutamic acid, and the activation of SAT1 induces lipid peroxidation. The overexpression of glutaminase 2 and SAT1 promotes PUFA oxidation and lipid peroxidation, leading to ferroptosis. The transcription target gene cyclin-dependent kinase inhibitor 1A is used to reduce GSH and ROS and delay ferroptosis (84). The specific mechanism by which P53 regulates ferroptosis needs further study.

#### 2.5.2 The Keap1-NRF2 pathway mediates ferroptosis

The NRF2 transcription factor stimulates the production of NADPH by blocking the expression of antioxidant genes and increasing the expression of enzymes in the pentose phosphate pathway, which decreases the sensitivity of cells to ferroptosis (85). Under normal physiological conditions, activation of the Keap1-NRF2 signaling pathway promotes the activation of System Xc - and the expression of GPX4 and accelerates cysteine glutamate transport, thereby clearing accumulated lipid peroxidation and inhibiting ferroptosis (86). Activation of NRF2 reduces iron absorption, limits ROS production, and enhances cellular antioxidant capacity (87). P62 strictly controls NRF2 and can inhibit ferroptosis. P62 is an autophagic receptor that directly inhibits Keap1 while promoting NRF2 activation (88). NRF2 regulates multiple targets, such as genes regulating GSH synthesis and encoding antioxidant proteins (89). The iron-chelating enzymes HO-1, FTH, and FTL are all strictly controlled by NRF2 (90). Glutamate cysteine ligase, GSH synthase, and SLC7A11 are also transcriptional targets of NRF2 (91).

### 2.6 The interaction between ferroptosis and other types of cell death

Ferroptosis may interact with other types of cell death (92). Anthraquinone modifications can significantly upregulate the expression of glucose-regulated protein 78, activate transcription factor 4, and downregulate the expression of the essential ferroptosis protein GPX4. Endoplasmic reticulum stress induces apoptosis and accompanies ferroptosis. The autophagy pathway can degrade ferritin, while ferroptosis regulatory proteins can regulate autophagy (93). Ferritin autophagy and lipid autophagy can promote ferroptosis by regulating iron metabolism and lipid peroxidation (94). Under oxidative stress, autophagy protects mitochondrial integrity by clearing ROS, preventing cell apoptosis, and exerting a protective effect (95). Excessive ROS-induced autophagy can also lead to cell death (96). Lipid peroxidation can attach to particular mitochondria and autophagy-related proteins, leading to autophagic cell death and cellular dysfunction (97). In summary, ferroptosis interacts with and promotes other types of cell death.

## 3 The role of ferroptosis in female reproductive disorders

### 3.1 The impact of ferroptosis on the process of follicular development

ROS and antioxidants in the ovaries play critical regulatory roles during oocyte maturation, fertilization, and embryonic development and implantation (98). Studies have shown that excessive iron in follicular fluid significantly increases ROS levels in mouse oocytes, leading to a decrease in the maturation rate of oocytes (99). Recurrent bleeding from ovarian lesions can lead to iron overload, increased ferroptosis, and decreased ovarian function, affecting follicular development and oocyte quality. ROS in follicles promotes apoptosis in most follicular cells (100). Studies have shown that blocking the production of GSH increases antral follicular atresia in rats (101). Research has shown that the expression of TF in early follicular atresia is significantly reduced, while the expression of the iron chaperone protein PCBP is increased considerably. During follicular development, Basonclin1 maintains lipid metabolism and redox homeostasis in oocytes. Research has confirmed abnormal levels and pathways of ferroptosis-related indicators in basonclin1-truncated mouse oocytes (102). Neurofibromin 2 expression was significantly reduced in basonclin1-deficient oocytes, while the expression levels of yes-associated protein, TFR, and ACSL4 increased considerably, triggering oocyte ferroptosis (103).

### 3.2 Ferroptosis of GCs leads to immature oocytes

Oogenesis is achieved through the interaction between the oocyte and the microenvironment of the follicle (104). The production of various cytokines and hormones in follicular fluid mainly relies on ovarian GCs, which play an essential role in oogenesis (105). Excessive iron in the internal environment can cause ferroptosis in ovarian GCs, which slows oocyte maturation and follicle development, increasing the risk of infertility (106, 107). Researchers have shown that a TFR-mediated increase in iron uptake in GCs induces the release of ROS, mitochondrial autophagy, and lipid peroxidation (108). The levels of FTH, TF, and TFRC in the ovarian GCs of infertile women are much lower than those in the ovarian GCs of healthy women (109). This finding suggested that FTH is a crucial regulator of ovarian follicle development and atresia (110). CircRHBG competes with SLC7A11 for binding to miR-515-5p, inhibiting GC ferroptosis. Therefore, the knockout of circRHBG can promote ferroptosis (111).

### 3.3 Oxidative stress impairs reproductive function

Mitochondrial dysfunction and excessive ROS production are characteristics of ferroptosis, and mitochondrial dysfunction further promotes ROS production, leading to oxidative stress, which induces the development and exacerbation of ferroptosis (112, 113). The other key indicator of ferroptosis is an increase in the levels of MDA, the final product of lipid oxidation (114). MDA can affect the activity of respiratory chain complexes and critical enzymes in mitochondria and aggravate membrane damage. A reduction in mitochondrial DNA is correlated with IR, hyperandrogenemia, and polycystic ovarian morphology in women with PCOS (115–117). Oxidative stress, ferroptosis, and PCOS interact with each other (118). An imbalance in total antioxidant levels in the serum of women with PCOS can exacerbate cell damage and reduce cellular defense ability (119). By comparing the oxidative stress indices of women with PCOS and healthy women, it was found that women with PCOS have significantly greater GPX and GSH reductase activities (120). The concentration of these substances directly affects the maturation and quality of oocytes, fertilization, and embryonic development (121). Metabolomic analysis of follicular fluid from clinical PCOS patients revealed mitochondrial dysfunction, oxidation−reduction potential imbalance, and increased oxidative stress in cumulus cells (122). The elevated levels of autoantibodies and ROS in the serum of PCOS patients suggest that oxidative stress may be one of the critical reasons for the abnormal endometrial environment in PCOS patients (123). Insulin is the primary regulator of oxidative phosphorylation, and its secretion can directly affect mitochondrial function (124, 125).

### 3.4 The impact of ferroptosis on pregnancy outcomes

Iron homeostasis plays an essential role in maintaining pregnancy. *In vitro* experimental studies have shown that appropriate iron in protein-free embryo culture medium can promote embryonic development, while iron overload is not conducive to embryonic development. Ferroptosis is associated with placental injury or miscarriage (126). During the abortion process, the large amount of ROS generated increases the generation of lipid peroxidation at the maternal-fetal interface, providing a primary condition for ferroptosis. Ferroptosis is triggered in the uterus and placenta of PCOS rats with hyperandrogenemia and IR. Compared with those in the control group, the expression levels of GPX4 and GSH in the uterus and placenta of PCOS rats were lower, and the expression levels of the ferroptosis-related genes ACSL4, TFCR1, SLC7A11, and glutamate cysteine ligase C were significantly increased (127). Ferroptosis is closely related to spontaneous preterm birth, and PLA2G6 can alleviate ferroptosis caused by GPX4 inhibition during pregnancy in mice (128).

## 4 Ferroptosis in PCOS

**Figure 3:**
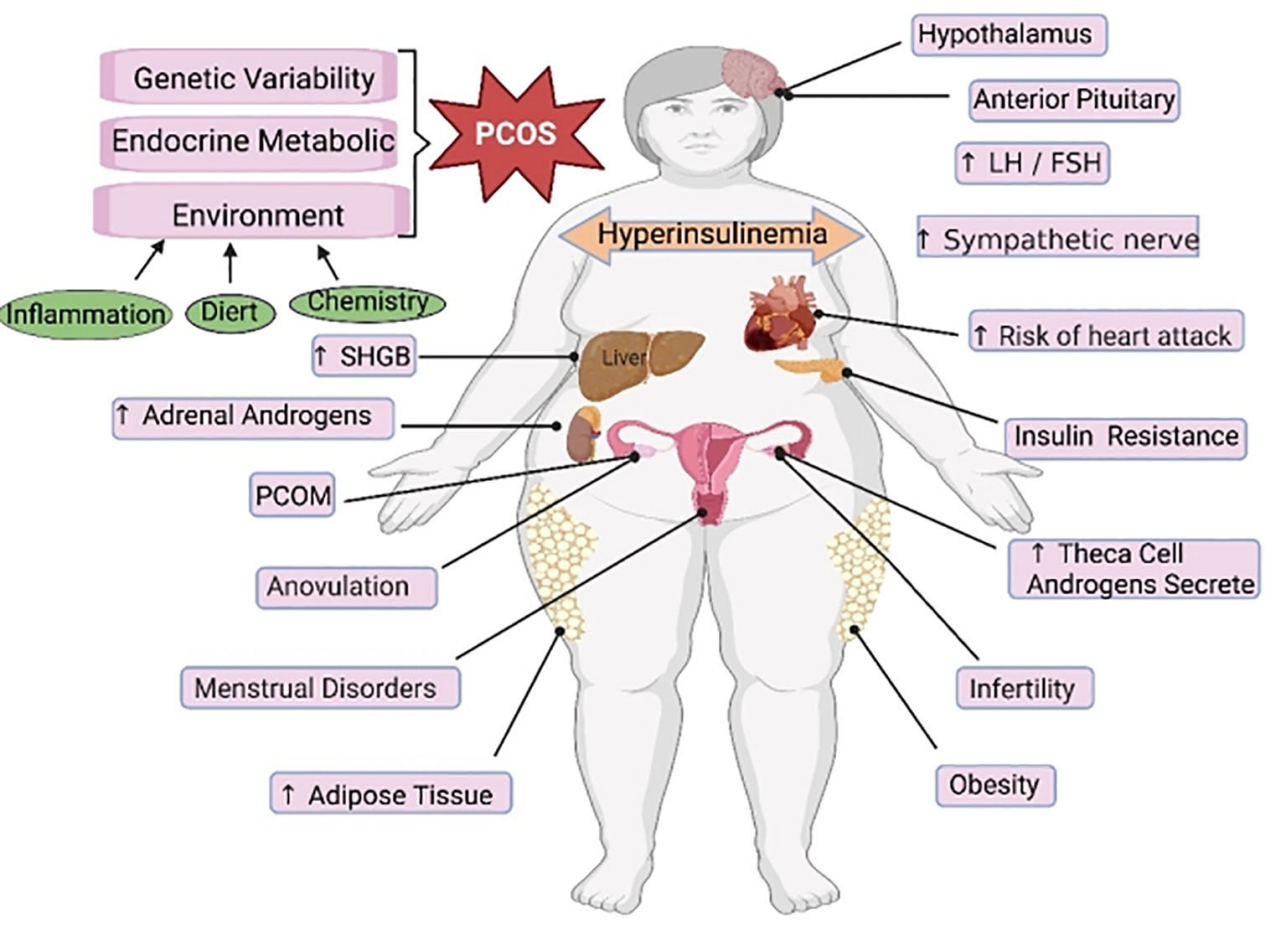
The main regulatory mechanisms of ferroptosis in PCOS. PCOS, polycystic ovarian syndrome; SHGB, sex hormone-binding globulin; LH, luteinizing hormone; FSH, follicle-stimulating hormone.

**Figure 4:**
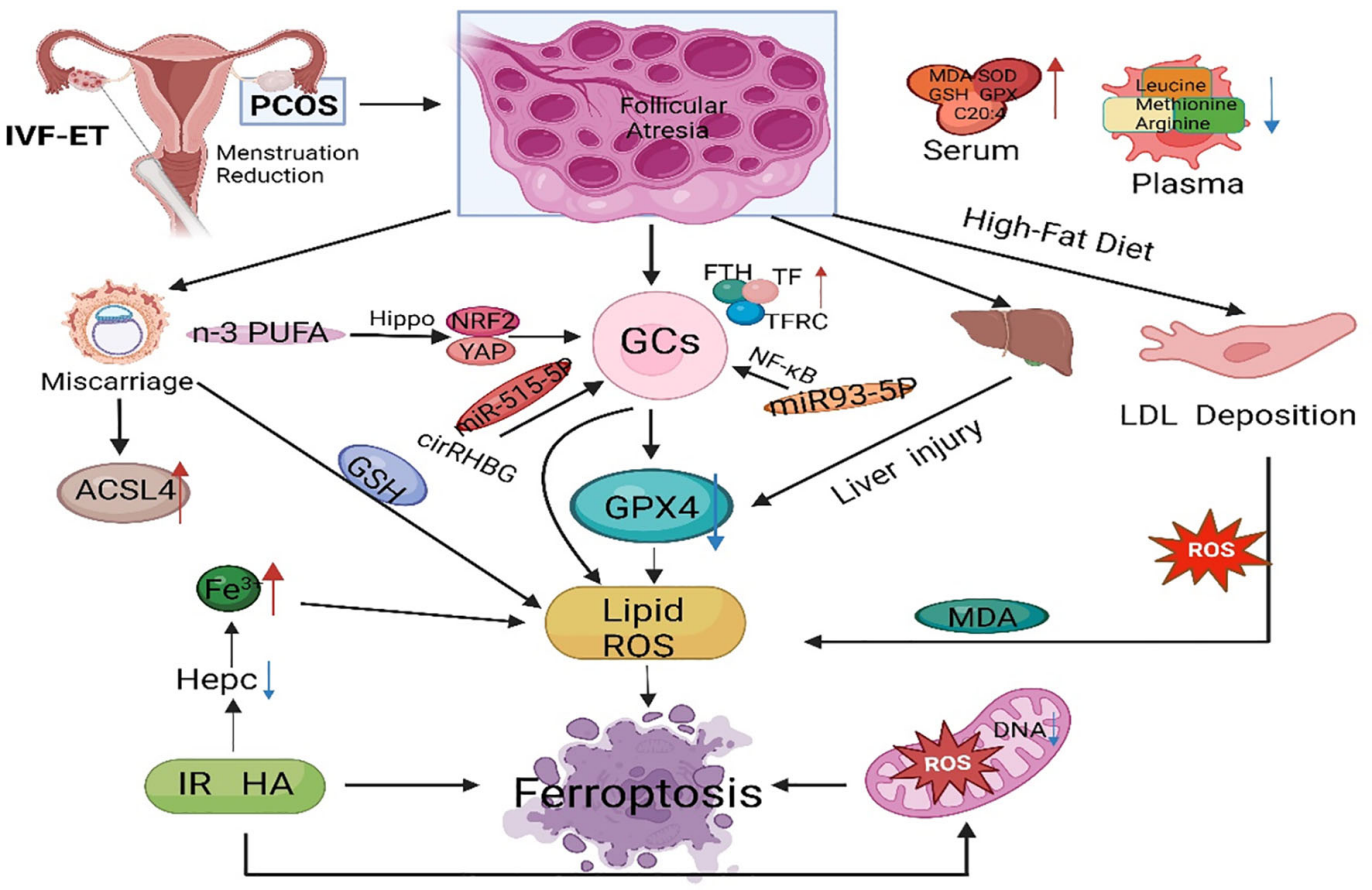
The main regulatory mechanisms of ferroptosis in PCOS. PCOS, polycystic ovarian syndrome; GCs, granulosa cells; GPX4, glutathione peroxidase 4; FTH, ferritin heavy chain; TF, transferrin; TFRC, transferrin receptor; IVF-ET, *in vitro* fertilization and embryo transfer; PUFA, polyunsaturated fatty acid; ACSL4, acyl-CoA synthetase long chain family member 4; IR, insulin resistance; ROS, reactive oxygen species; MDA, malondialdehyde; GSH, glutathione; NRF2, nuclear factor-erythroid 2-related factor 2.

PCOS is a reproductive endocrine disorder that not only affects women’s reproductive and physiological health but also leads to complications such as diabetes mellitus type 2 (T2DM), obesity, familial cardiovascular disease, and cardiovascular disease (129). PCOS has transcended the field of obstetrics and gynecology and affects various significant systems throughout the body, seriously threatening women’s physical and mental health, affecting their quality of life, and causing lifelong endocrine and metabolic diseases (130, 131) (**Figure 3**). The etiology of PCOS has not yet been clarified, although much work has been done on this topic over the past few decades (132). The onset of PCOS involves multiple factors, such as hyperandrogenemia, IR, and abnormal follicular development (133–135). Ferroptosis affects the proliferation and apoptosis of granulosa cells (GCs), affecting endocrine and metabolic processes (**Figure 4**).

Iron is an essential trace element for life and an important component of hemoglobin, myoglobin, and various enzymes (136). Iron participates in multiple critical physiological and biochemical processes in the body, including oxygen transport, DNA biosynthesis, and ATP synthesis (137). Research has shown that regardless of obesity, the serum Fn levels in PCOS patients are significantly greater than those in control individuals, indicating that abnormal iron metabolism may be involved in the occurrence and development of PCOS (138). Iron overload occurs in PCOS patients, potentially due to chronic oligomenorrhea and decreased hepcidin concentration (139). Research has shown that the serum Fn concentration is directly proportional to the severity of menstrual dysfunction, suggesting that the iron retention effect of chronic oligomenorrhea may be associated with increased iron storage in some PCOS patients (140). The compensatory hyperinsulinemia caused by PCOS may promote iron absorption (141, 142). Additionally, with the combined action of IR and excessive androgen in PCOS patients, TF inhibition is decreased, iron absorption in the intestine is increased, and iron release from macrophages is decreased, leading to iron overload (143).

### 4.1 Ferroptosis is involved in endocrine and metabolic disorders in PCOS

Iron overload can affect glucose metabolism, leading to IR and exacerbating metabolic abnormalities in PCOS patients (144). On the one hand, iron can affect the secretion and sensitivity of insulin and inhibit liver glycogen production, and insulin secretion decreases with increasing liver iron storage, leading to systemic hyperinsulinemia. On the other hand, insulin can promote iron absorption and Fn synthesis while also promoting glucose transport. Excessive androgen and insulin resistance can activate ferroptosis in the uterus and placenta of pregnant women with PCOS (145). The plasma levels of leucine, isoleucine, methionine, glutamine, and arginine were much lower in PCOS patients than in healthy controls (146). Compared to those in healthy women, serum bioactive lipid levels are much lower in women with PCOS (147). Arachidonic acid levels in the serum of PCOS rats were significantly greater than those in the serum of control rats (148). Phosphatidylethanolamine combined with arachidonic acid is an essential phospholipid that causes ferroptosis, which means that there are appropriate conditions for ferroptosis in PCOS patients (149).

### 4.2 Oxidative stress mediates metabolic abnormalities in PCOS

Oxidative stress is closely related to obesity, IR, and HA in PCOS, and the induced apoptosis of ovarian GCs leads to follicular atresia, which is one of the mechanisms of ovulation disorders in PCOS (150). SOD is an important antioxidant enzyme for scavenging oxygen-free radicals and catalyzes the dismutation reaction of ROS to eliminate oxygen-free radicals and reduce damage to ovarian cells (151). MDA levels are positively correlated with BMI, triglyceride levels, low-density lipoprotein levels, systolic blood pressure, diastolic blood pressure, and HOMA-IR (152). Research has shown that ROS produced by monocytes in the pancreas plays an essential role in the abnormal development of cells (153). ROS-induced NF-κB can enter the nucleus and bind to chromatin to promote tumor necrosis factor-α (TNF-α) (154). Transcription can activate the PI3K/Akt/mTOR pathway to inhibit insulin secretion (155). In PCOS animal models, free fatty acids in ovarian tissue increase, glucose oxidation is inhibited, free fatty acid oxidation is enhanced, and ROS increase through the TCA cycle, leading to IR (156).

### 4.3 Ferroptosis and a high-fat diet

Obesity is closely related to PCOS and plays a crucial role in the occurrence and development of PCOS (157). In obese PCOS patients, long-term high-fat diets can induce inflammatory reactions and oxidative damage and exacerbate iron deposition, which is an essential factor influencing ferroptosis (158). A long-term high-fat diet can cause low-density lipoprotein, which is deposited in the endothelium of tissues. The prolonged accumulation of low-density lipoprotein can induce an oxidative stress response (159). Oxidative stress is one of the pathogenic mechanisms of PCOS, and many studies have confirmed that oxidative stress indicators are positively correlated with obesity. Compared with those in nonobese patients, the serum levels of the antioxidant substances MDA, SOD, GSH, and GPX were significantly greater in the obese PCOS group (160). In the environment of mature follicles, oxidative damage can further trigger chronic inflammatory reactions in the ovaries. ROS plays a crucial role in follicular development disorders (161). In follicular fluid containing high levels of ROS, a large amount of highly toxic MDA is produced, which can exacerbate cell damage in the ovaries. Therefore, obesity, ferroptosis, and the development of PCOS are closely related.

## 5 Ferroptosis contributes to the diagnosis and treatment of PCOS

Follicular development is a complex biological process of continuous change involving multiple hormones and regulatory factors, and the loss of oocytes from the ovary is irreversible (162). Abnormal follicular development can cause a wide range of female disorders and lead to reduced female fertility (163). Early preventive measures could mitigate the development of PCOS (164). The substances that regulate ferroptosis can serve as targets for diagnosing and treating PCOS, exhibiting broad research prospects. Zheng et al. reported that unexplained liver injury in PCOS patients and animal models was accompanied by increased Fe deposition and downregulation of hepcidin and GPX4 expression in the liver, indicating the importance of iron metabolism in this type of unexplained liver injury (165). Tang et al. reported that NEDD4L promotes ferroptosis in GCs and promotes the occurrence of PCOS by promoting GPX4 ubiquitination and degradation. NEDD4L decreased the viability of KGN cells and increased the levels of MDA and ROS. Moreover, ferroptosis inhibitors can block NEDD4L-induced KGN cell death, suggesting that NEDD4L regulates ferroptosis in KGN cells (166). Jiang et al. reported that KGN cells treated with DHEA exhibited ferroptosis characterized by decreased viability, inhibited GPX4 and SLC7A11 expression, increased ACSL4 expression, increased MDA levels and ROS accumulation, and increased lipid peroxidation. These findings may provide new insights into the pathophysiology and treatment of PCOS (167).

Zhang et al. noted that differentially expressed ferroptosis-related genes are associated with reproductive outcomes in infertile POCS patients, and they constructed a FerSig risk prognosis model (168). Lin et al. identified five essential differentially expressed ferroptosis-related genes (NOX1, ACVR1B, PHF21A, FTL, and GALNT14) that may be related to the pathogenesis of PCOS, providing a new perspective for the clinical diagnosis and treatment of PCOS (169). Research has shown that miR-93-5p regulates the NF-κB signaling pathway and promotes apoptosis and ferroptosis in GCs (170). Silencing miR-93-5p can prevent GC dysfunction and provide new molecular targets for diagnosing and treating PCOS. N-3 PUFAs activate Hippo, promote yes-associated protein 1 exocytosis, weaken cross-talk between yes-associated protein 1 and Nrf2, and ultimately activate ferroptosis sensitivity in ovarian GCs. N-3 PUFAs also inhibit excessive proliferation of GCs in ovarian follicles, by which n-3 PUFAs weaken PCOS, and identifying yes-associated protein 1 (Nrf2) as a potential therapeutic target for regulating GCs in PCOS (171). Transferrin receptor-mediated ROS promote ferroptosis in KGN cells by regulating NADPH oxidase 1/PTEN-induced kinase 1/acyl-CoA synthetase long-chain family member 4 signaling, and the inhibitory effects of TFRC/NOX1/PINK1/ACSL4 signaling on folliculogenesis could be a potential target for PCOS treatment (172). Peng et al. reported that metformin regulates ferroptosis through the SIRT3/AMPK/mTOR pathway to improve weight, metabolic disorders, and ovarian dysfunction in PCOS mice (173). Therefore, exploring the role of ferroptosis in the occurrence and development of PCOS can provide more ideas for mechanistic research on PCOS and more potential targets for PCOS treatment.

## 6 Conclusions

In recent years, substantial progress has been made in exploring ferroptosis. The regulatory network of iron homeostasis, lipid metabolism, amino acid metabolism, and antioxidant pathways provides new ideas for diagnosing and treating human diseases. Ferroptosis is expected to become a new biomarker for the development, treatment efficacy, and prognostic evaluation of PCOS. Research on ferroptosis in PCOS is still lacking. Further research on PCOS animal models with larger sample sizes is needed to validate the potential effects of ferroptosis on ovarian GCs, follicles, ovaries, and even the entire female reproductive system. More clinical studies are urgently needed. Assessing whether ferroptosis and its related molecules play essential roles in infertility, metabolic abnormalities, and other aspects of clinical PCOS and whether the administration of antioxidants can prevent ferroptosis, lipid peroxidation, and adverse maternal and fetal outcomes caused by maternal hyperandrogenemia and IR will provide insights and directions for future clinical diagnosis and treatment. Based on existing prevention and treatment methods for PCOS, interventions targeting different nodes of the ferroptosis regulatory network combined with other treatment methods for various patient etiologies, treatments, and prognoses are expected to be effective and reasonable in the future, thus achieving personalized treatment of PCOS.

## Author contributions

MW: Conceptualization, Formal analysis, Methodology, Project administration, Resources, Writing – original draft, Writing – review & editing. B-QZ: Conceptualization, Project administration, Resources, Supervision, Validation, Visualization, Writing – review & editing. SM: Conceptualization, Data curation, Formal analysis, Funding acquisition, Methodology, Resources, Writing – review & editing. YX: Formal analysis, Funding acquisition, Investigation, Methodology, Software, Writing – review & editing. D-HZ: Conceptualization, Data curation, Funding acquisition, Investigation, Methodology, Resources, Writing – original draft. J-SZ: Conceptualization, Data curation, Formal analysis, Funding acquisition, Software, Writing – review & editing. C-JL: Conceptualization, Data curation, Formal analysis, Methodology, Project administration, Resources, Visualization, Writing – review & editing. XZ: Conceptualization, Data curation, Investigation, Methodology, Supervision, Validation, Writing – review & editing. L-WZ: Conceptualization, Investigation, Supervision, Validation, Visualization, Writing – review & editing.

## Glossary

**Table:**

PCOS	polycystic ovarian syndrome
IR	insulin resistance
PUFAs	poly-unsaturated fatty acids
GPX4	glutathione peroxidase 4
ROS	reactive oxygen species
TF	transferrin
TFCR1	transferrin receptor 1
FTH	ferritin heavy chain
FTL	ferritin light chain
Fn	ferritin
FPN	ferroportin
NCOA4	nuclear receptor coactivator 4
ATG5	autophagy-related genes 5
ACSL4	acyl-CoA synthetase long chain family member 4
GSH	consumption of glutathione
System Xc -	the cystine/glutamic acid reverse transporter
SLC7A11	solute carrier family 7 member 11
NRF2	nuclear factor-erythroid 2-related factor 2
HO-1	heme oxygenase-1
MDA	malondialdehyde
FSP1	ferroptosis suppressor protein 1
BH4	tetrahydrobiopterin
SAT1	spermidine/spermine N1-acetyltransferase 1
T2DM	type 2 diabetes mellitus
GCs	granulosa cells
SOD	superoxide dismutase
TNF-α	tumor necrosis factor-α

## References

[1] Bell RJ Islam RM Skiba MA Davis SR. Reply: A single cut-off value of anti-Mullerian hormone should not be used for the diagnosis of PCOS in all reproductive-aged women. Hum Reprod. (2022) 37:622. doi: 10.1093/humrep/deac013 35106571

[2] Ehrmann DA. Polycystic ovary syndrome. N Engl J Med. (2005) 352:1223–36. doi: 10.1056/NEJMra041536 15788499

[3] Essah PA Nestler JE. The metabolic syndrome in polycystic ovary syndrome. J Endocrinol Invest. (2006) 29:270–80. doi: 10.1007/BF03345554 16682845

[4] Dapas M Dunaif A. Deconstructing a syndrome: genomic insights into PCOS causal mechanisms and classification. Endocr Rev. (2022) 43:927–65. doi: 10.1210/endrev/bnac001 PMC9695127 35026001

[5] Capelletti MM Manceau H Puy H Peoc’h K. Ferroptosis in liver diseases: an overview. Int J Mol Sci. (2020) 21(14):4908. doi: 10.3390/ijms21144908 32664576 PMC7404091

[6] Fernandez-Real JM Lopez-Bermejo A Ricart W. Cross-talk between iron metabolism and diabetes. Diabetes. (2002) 51:2348–54. doi: 10.2337/diabetes.51.8.2348 12145144

[7] Cooney LG Lee I Sammel MD Dokras A. High prevalence of moderate and severe depressive and anxiety symptoms in polycystic ovary syndrome: a systematic review and meta-analysis. Hum Reprod. (2017) 32:1075–91. doi: 10.1093/humrep/dex044 28333286

[8] Walters KA Bertoldo MJ Handelsman DJ. Evidence from animal models on the pathogenesis of PCOS. Best Pract Res Clin Endocrinol Metab. (2018) 32:271–81. doi: 10.1016/j.beem.2018.03.008 29779581

[9] Tay CT Teede HJ Hill B Loxton D Joham AE. Increased prevalence of eating disorders, low self-esteem, and psychological distress in women with polycystic ovary syndrome: a community-based cohort study. Fertil Steril. (2019) 112:353–61. doi: 10.1016/j.fertnstert.2019.03.027 31056307

[10] Friedmann Angeli JP Schneider M Proneth B Tyurina YY Tyurin VA Hammond VJ. Inactivation of the ferroptosis regulator Gpx4 triggers acute renal failure in mice. Nat Cell Biol. (2014) 16:1180–91. doi: 10.1038/ncb3064 PMC4894846 25402683

[11] Lei G Zhuang L Gan B. Targeting ferroptosis as a vulnerability in cancer. Nat Rev Cancer. (2022) 22:381–96. doi: 10.1038/s41568-022-00459-0 PMC10243716 35338310

[12] Banuls C Rovira-Llopis S Martinez de Maranon A Veses S Jover A Gomez M. Metabolic syndrome enhances endoplasmic reticulum, oxidative stress, and leukocyte-endothelium interactions in PCOS. Metabolism. (2017) 71:153–62. doi: 10.1016/j.metabol.2017.02.012 28521868

[13] Lai Q Xiang W Li Q Zhang H Li Y Zhu G. Oxidative stress in granulosa cells contributes to poor oocyte quality and IVF-ET outcomes in women with polycystic ovary syndrome. Front Med. (2018) 12:518–24. doi: 10.1007/s11684-017-0575-y 29260383

[14] Gao M Yi J Zhu J Minikes AM Monian P Thompson CB. Role of mitochondria in ferroptosis. Mol Cell. (2019) 73:354–63 e3. doi: 10.1016/j.molcel.2018.10.042 30581146 PMC6338496

[15] Yan B Ai Y Sun Q Ma Y Cao Y Wang J. Membrane damage during ferroptosis is caused by oxidation of phospholipids catalyzed by the oxidoreductases POR and CYB5R1. Mol Cell. (2021) 81:355–69 e10. doi: 10.1016/j.molcel.2020.11.024 33321093

[16] Nasri F Zare M Doroudchi M Gharesi-Fard B. Proteome analysis of CD4(+) T cells reveals differentially expressed proteins in infertile polycystic ovary syndrome patients. Endocr Metab Immune Disord Drug Targets. (2021) 21:1998–2004. doi: 10.2174/1871530320666201119152323 33213354

[17] Dixon SJ Lemberg KM Lamprecht MR Skouta R Zaitsev EM Gleason CE. Ferroptosis: an iron-dependent form of nonapoptotic cell death. Cell. (2012) 149:1060–72. doi: 10.1016/j.cell.2012.03.042 PMC3367386 22632970

[18] Liu Y Zhou L Xu Y Li K Zhao Y Qiao H. Heat shock proteins and ferroptosis. Front Cell Dev Biol. (2022) 10:864635.doi: 10.3389/fcell.2022.864635 35478955 PMC9035830

[19] Wen J Chen H Ren Z Zhang P Chen J Jiang S. Ultrasmall iron oxide nanoparticles induced ferroptosis via Beclin1/ATG5-dependent autophagy pathway. Nano Converg. (2021) 8:10. doi: 10.1186/s40580-021-00260-z 33796911 PMC8017028

[20] D’Herde K Krysko DV. Ferroptosis: Oxidized PEs trigger death. Nat Chem Biol. (2017) 13:4–5. doi: 10.1038/nchembio.2261 27842067

[21] Yang WS Kim KJ Gaschler MM Patel M Shchepinov MS Stockwell BR. Peroxidation of poly-unsaturated fatty acids by lipoxygenases drives ferroptosis. Proc Natl Acad Sci USA. (2016) 113:E4966–75. doi: 10.1073/pnas.1603244113 PMC5003261 27506793

[22] Li C Deng X Zhang W Xie X Conrad M Liu Y. Novel allosteric activators for ferroptosis regulator glutathione peroxidase 4. J Med Chem. (2019) 62:266–75. doi: 10.1021/acs.jmedchem.8b00315 29688708

[23] Forcina GC Dixon SJ. GPX4 at the crossroads of lipid homeostasis and ferroptosis. Proteomics. (2019) 19:e1800311. doi: 10.1002/pmic.201800311 30888116

[24] Zou Y Henry WS Ricq EL Graham ET Phadnis VV Maretich P. Plasticity of ether lipids promotes ferroptosis susceptibility and evasion. Nature. (2020) 585:603–8. doi: 10.1038/s41586-020-2732-8 PMC8051864 32939090

[25] Zou Y Li H Graham ET Deik AA Eaton JK Wang W. Cytochrome P450 oxidoreductase contributes to phospholipid peroxidation in ferroptosis. Nat Chem Biol. (2020) 16:302–9. doi: 10.1038/s41589-020-0472-6 PMC7353921 32080622

[26] Agmon E Solon J Bassereau P Stockwell BR. Modelling the effects of lipid peroxidation during ferroptosis on membrane properties. Sci Rep. (2018) 8:5155. doi: 10.1038/s41598-018-23408-0 29581451 PMC5979948

[27] Hadian K Stockwell BR. SnapShot: ferroptosis. Cell. (2020) 181:1188– e1. doi: 10.1016/j.cell.2020.04.039 PMC8157339 32470402

[28] Stockwell BR Jiang X Gu W. Emerging mechanisms and disease relevance of ferroptosis. Trends Cell Biol. (2020) 30:478–90. doi: 10.1016/j.tcb.2020.02.009 PMC7230071 32413317

[29] Salnikow K. Role of iron in cancer. Semin Cancer Biol. (2021) 76:189–94. doi: 10.1016/j.semcancer.2021.04.001 33901632

[30] Stockwell BR Friedmann Angeli JP Bayir H Bush AI Conrad M Dixon SJ. Ferroptosis: A regulated cell death nexus linking metabolism, redox biology, and disease. Cell. (2017) 171:273–85. doi: 10.1016/j.cell.2017.09.021 PMC5685180 28985560

[31] Kuang F Liu J Tang D Kang R. Oxidative damage and antioxidant defense in ferroptosis. Front Cell Dev Biol. (2020) 8:586578doi: 10.3389/fcell.2020.586578 33043019 PMC7527737

[32] Cepelak I Dodig S Dodig DC. Ferroptosis: regulated cell death. Arh Hig Rada Toksikol. (2020) 71:99–109. doi: 10.2478/aiht-2020-71-3366 32975106 PMC7968485

[33] Chen PH Wu J Ding CC Lin CC Pan S Bossa N. Kinome screen of ferroptosis reveals a novel role of ATM in regulating iron metabolism. Cell Death Differ. (2020) 27:1008–22. doi: 10.1038/s41418-019-0393-7 PMC7206124 31320750

[34] Yang WS SriRamaratnam R Welsch ME Shimada K Skouta R Viswanathan VS. Regulation of ferroptotic cancer cell death by GPX4. Cell. (2014) 156:317–31. doi: 10.1016/j.cell.2013.12.010 PMC4076414 24439385

[35] Galaris D Barbouti A Pantopoulos K. Iron homeostasis and oxidative stress: An intimate relationship. Biochim Biophys Acta Mol Cell Res. (2019) 1866:118535. doi: 10.1016/j.bbamcr.2019.118535 31446062

[36] Turcu AL Versini A Khene N Gaillet C Caneque T Muller S. DMT1 inhibitors kill cancer stem cells by blocking lysosomal iron translocation. Chemistry. (2020) 26:7369–73. doi: 10.1002/chem.202000159 32083771

[37] Rockfield S Raffel J Mehta R Rehman N Nanjundan M. Iron overload and altered iron metabolism in ovarian cancer. Biol Chem. (2017) 398:995–1007. doi: 10.1515/hsz-2016-0336 28095368 PMC5545069

[38] Pantopoulos K Porwal SK Tartakoff A Devireddy L. Mechanisms of mammalian iron homeostasis. Biochemistry. (2012) 51:5705–24. doi: 10.1021/bi300752r PMC3572738 22703180

[39] Angeli JPF Shah R Pratt DA Conrad M. Ferroptosis inhibition: mechanisms and opportunities. Trends Pharmacol Sci. (2017) 38:489–98. doi: 10.1016/j.tips.2017.02.005 28363764

[40] Xie Y Hou W Song X Yu Y Huang J Sun X. Ferroptosis: process and function. Cell Death Differ. (2016) 23:369–79. doi: 10.1038/cdd.2015.158 PMC5072448 26794443

[41] Gao M Monian P Pan Q Zhang W Xiang J Jiang X. Ferroptosis is an autophagic cell death process. Cell Res. (2016) 26:1021–32. doi: 10.1038/cr.2016.95 PMC5034113 27514700

[42] Park E Chung SW. ROS-mediated autophagy increases intracellular iron levels and ferroptosis by ferritin and transferrin receptor regulation. Cell Death Dis. (2019) 10:822. doi: 10.1038/s41419-019-2064-5 31659150 PMC6817894

[43] Mancias JD Wang X Gygi SP Harper JW Kimmelman AC. Quantitative proteomics identifies NCOA4 as the cargo receptor mediating ferritinophagy. Nature. (2014) 509:105–9. doi: 10.1038/nature13148 PMC4180099 24695223

[44] Gryzik M Asperti M Denardo A Arosio P Poli M. NCOA4-mediated ferritinophagy promotes ferroptosis induced by erastin, but not by RSL3 in HeLa cells. Biochim Biophys Acta Mol Cell Res. (2021) 1868:118913. doi: 10.1016/j.bbamcr.2020.118913 33245979

[45] Kang B Jiang D Ma R He H. Evidence for a role of ferritin heavy chain in mediating reproductive processes of geese. Reprod Biol. (2015) 15:205–9. doi: 10.1016/j.repbio.2015.10.001 26679160

[46] Tang LJ Zhou YJ Xiong XM Li NS Zhang JJ Luo XJ. Ubiquitin-specific protease 7 promotes ferroptosis via activation of the p53/TFCR1 pathway in the rat hearts after ischemia/reperfusion. Free Radic Biol Med. (2021) 162:339–52. doi: 10.1016/j.freeradbiomed.2020.10.307 33157209

[47] Dixon SJ Stockwell BR. The role of iron and reactive oxygen species in cell death. Nat Chem Biol. (2014) 10:9–17. doi: 10.1038/nchembio.1416 24346035

[48] Green DR. The coming decade of cell death research: five riddles. Cell. (2019) 177:1094–107. doi: 10.1016/j.cell.2019.04.024 PMC6534278 31100266

[49] Shimada K Skouta R Kaplan A Yang WS Hayano M Dixon SJ. Global survey of cell death mechanisms reveals metabolic regulation of ferroptosis. Nat Chem Biol. (2016) 12:497–503. doi: 10.1038/nchembio.2079 27159577 PMC4920070

[50] Wang L Liu Y Du T Yang H Lei L Guo M. ATF3 promotes erastin-induced ferroptosis by suppressing System Xc -(). Cell Death Differ. (2020) 27:662–75. doi: 10.1038/s41418-019-0380-z PMC7206049 31273299

[51] Kagan VE Mao G Qu F Angeli JP Doll S Croix CS. Oxidized arachidonic and adrenic PEs navigate cells to ferroptosis. Nat Chem Biol. (2017) 13:81–90. doi: 10.1038/nchembio.2238 27842066 PMC5506843

[52] Yuan H Li X Zhang X Kang R Tang D. Identification of ACSL4 as a biomarker and contributor of ferroptosis. Biochem Biophys Res Commun. (2016) 478:1338–43. doi: 10.1016/j.bbrc.2016.08.124 27565726

[53] Dixon SJ Winter GE Musavi LS Lee ED Snijder B Rebsamen M. Human haploid cell genetics reveals roles for lipid metabolism genes in nonapoptotic cell death. ACS Chem Biol. (2015) 10:1604–9. doi: 10.1021/acschembio.5b00245 PMC4509420 25965523

[54] Doll S Proneth B Tyurina YY Panzilius E Kobayashi S Ingold I. ACSL4 dictates ferroptosis sensitivity by shaping cellular lipid composition. Nat Chem Biol. (2017) 13:91–8. doi: 10.1038/nchembio.2239 PMC5610546 27842070

[55] Enke U Seyfarth L Schleussner E Markert UR. Impact of PUFA on early immune and fetal development. Br J Nutr. (2008) 100:1158–68. doi: 10.1017/S000711450801413X 18590581

[56] Yang WS Stockwell BR. Ferroptosis: death by lipid peroxidation. Trends Cell Biol. (2016) 26:165–76. doi: 10.1016/j.tcb.2015.10.014 PMC4764384 26653790

[57] Hassannia B Vandenabeele P Vanden Berghe T. Targeting ferroptosis to iron out cancer. Cancer Cell. (2019) 35:830–49. doi: 10.1016/j.ccell.2019.04.002 31105042

[58] Kinowaki Y Kurata M Ishibashi S Ikeda M Tatsuzawa A Yamamoto M. Glutathione peroxidase 4 overexpression inhibits ROS-induced cell death in diffuse large B-cell lymphoma. Lab Invest. (2018) 98:609–19. doi: 10.1038/s41374-017-0008-1 29463878

[59] Sbodio JI Snyder SH Paul BD. Regulators of the transsulfuration pathway. Br J Pharmacol. (2019) 176:583–93. doi: 10.1111/bph.14446 PMC6346075 30007014

[60] Sun Y Chen P Zhai B Zhang M Xiang Y Fang J. The emerging role of ferroptosis in inflammation. BioMed Pharmacother. (2020) 127:110108. doi: 10.1016/j.biopha.2020.110108 32234642

[61] Kobayashi S Hamashima S Homma T Sato M Kusumi R Bannai S. Cystine/glutamate transporter, system x(c)(-), is involved in nitric oxide production in mouse peritoneal macrophages. Nitric Oxide. (2018) 78:32–40. doi: 10.1016/j.niox.2018.05.005 29792932

[62] Vaskova J Kocan L Vasko L Perjesi P. Glutathione-related enzymes and proteins: A review. Molecules. (2023) 28(3):1447. doi: 10.3390/molecules28031447 36771108 PMC9919958

[63] McBean GJ. The transsulfuration pathway: a source of cysteine for glutathione in astrocytes. Amino Acids. (2012) 42:199–205. doi: 10.1007/s00726-011-0864-8 21369939

[64] Hao S Yu J He W Huang Q Zhao Y Liang B. Cysteine dioxygenase 1 mediates erastin-induced ferroptosis in human gastric cancer cells. Neoplasia. (2017) 19:1022–32. doi: 10.1016/j.neo.2017.10.005 PMC5686465 29144989

[65] Gao M Monian P Quadri N Ramasamy R Jiang X. Glutaminolysis and transferrin regulate ferroptosis. Mol Cell. (2015) 59:298–308. doi: 10.1016/j.molcel.2015.06.011 26166707 PMC4506736

[66] Koppula P Zhang Y Zhuang L Gan B. Amino acid transporter SLC7A11/xCT at the crossroads of regulating redox homeostasis and nutrient dependency of cancer. Cancer Commun (Lond). (2018) 38:12. doi: 10.1186/s40880-018-0288-x 29764521 PMC5993148

[67] Ye Z Liu W Zhuo Q Hu Q Liu M Sun Q. Ferroptosis: Final destination for cancer? Cell Prolif. (2020) 53:e12761. doi: 10.1111/cpr.12761 32100402 PMC7106955

[68] Song X Zhu S Chen P Hou W Wen Q Liu J. AMPK-mediated BECN1 phosphorylation promotes ferroptosis by directly blocking system X(c)(-) activity. Curr Biol. (2018) 28:2388–99 e5. doi: 10.1016/j.cub.2018.05.094 30057310 PMC6081251

[69] Dong H Qiang Z Chai D Peng J Xia Y Hu R. Nrf2 inhibits ferroptosis and protects against acute lung injury due to intestinal ischemia reperfusion via regulating SLC7A11 and HO-1. Aging (Albany NY). (2020) 12:12943–59. doi: 10.18632/aging.v12i13 PMC7377827 32601262

[70] Tang D Chen X Kang R Kroemer G. Ferroptosis: molecular mechanisms and health implications. Cell Res. (2021) 31:107–25. doi: 10.1038/s41422-020-00441-1 PMC8026611 33268902

[71] Latunde-Dada GO. Ferroptosis: Role of lipid peroxidation, iron and ferritinophagy. Biochim Biophys Acta Gen Subj. (2017) 1861:1893–900. doi: 10.1016/j.bbagen.2017.05.019 28552631

[72] Seibt TM Proneth B Conrad M. Role of GPX4 in ferroptosis and its pharmacological implication. Free Radic Biol Med. (2019) 133:144–52. doi: 10.1016/j.freeradbiomed.2018.09.014 30219704

[73] Goji T Takahara K Negishi M Katoh H. Cystine uptake through the cystine/glutamate antiporter xCT triggers glioblastoma cell death under glucose deprivation. J Biol Chem. (2017) 292:19721–32. doi: 10.1074/jbc.M117.814392 PMC5712613 29038291

[74] Ingold I Berndt C Schmitt S Doll S Poschmann G Buday K. Selenium utilization by GPX4 is required to prevent hydroperoxide-induced ferroptosis. Cell. (2018) 172:409–22.e21. doi: 10.1016/j.cell.2017.11.048 29290465

[75] Jiang X Stockwell BR Conrad M. Ferroptosis: mechanisms, biology and role in disease. Nat Rev Mol Cell Biol. (2021) 22:266–82. doi: 10.1038/s41580-020-00324-8 PMC8142022 33495651

[76] Conrad M Sato H. The oxidative stress-inducible cystine/glutamate antiporter, system x (c) (-): cystine supplier and beyond. Amino Acids. (2012) 42:231–46. doi: 10.1007/s00726-011-0867-5 21409388

[77] Doll S Freitas FP Shah R Aldrovandi M da Silva MC Ingold I. FSP1 is a glutathione-independent ferroptosis suppressor. Nature. (2019) 575:693–8. doi: 10.1038/s41586-019-1707-0 31634899

[78] Bersuker K Hendricks JM Li Z Magtanong L Ford B Tang PH. The CoQ oxidoreductase FSP1 acts parallel to GPX4 to inhibit ferroptosis. Nature. (2019) 575:688–92. doi: 10.1038/s41586-019-1705-2 PMC6883167 31634900

[79] Kraft VAN Bezjian CT Pfeiffer S Ringelstetter L Muller C Zandkarimi F. GTP cyclohydrolase 1/tetrahydrobiopterin counteract ferroptosis through lipid remodeling. ACS Cent Sci. (2020) 6:41–53. doi: 10.1021/acscentsci.9b01063 31989025 PMC6978838

[80] Bridges R Lutgen V Lobner D Baker DA. Thinking outside the cleft to understand synaptic activity: contribution of the cystine-glutamate antiporter (System Xc-) to normal and pathological glutamatergic signaling. Pharmacol Rev. (2012) 64:780–802. doi: 10.1124/pr.110.003889 22759795 PMC3400835

[81] Mao C Liu X Zhang Y Lei G Yan Y Lee H. DHODH-mediated ferroptosis defence is a targetable vulnerability in cancer. Nature. (2021) 593:586–90. doi: 10.1038/s41586-021-03539-7 PMC8895686 33981038

[82] Liu J Zhang C Wang J Hu W Feng Z. The Regulation of Ferroptosis by Tumor Suppressor p53 and its pathway. Int J Mol Sci. (2020) 21(21):8387. doi: 10.3390/ijms21218387 33182266 PMC7664917

[83] Jiang L Kon N Li T Wang SJ Su T Hibshoosh H. Ferroptosis as a p53-mediated activity during tumour suppression. Nature. (2015) 520:57–62. doi: 10.1038/nature14344 25799988 PMC4455927

[84] Tarangelo A Magtanong L Bieging-Rolett KT Li Y Ye J Attardi LD. p53 suppresses metabolic stress-induced ferroptosis in cancer cells. Cell Rep. (2018) 22:569–75. doi: 10.1016/j.celrep.2017.12.077 PMC5791910 29346757

[85] Shimada K Hayano M Pagano NC Stockwell BR. Cell-line selectivity improves the predictive power of pharmacogenomic analyses and helps identify NADPH as biomarker for ferroptosis sensitivity. Cell Chem Biol. (2016) 23:225–35. doi: 10.1016/j.chembiol.2015.11.016 PMC4792701 26853626

[86] Sun X Ou Z Chen R Niu X Chen D Kang R. Activation of the p62-Keap1-NRF2 pathway protects against ferroptosis in hepatocellular carcinoma cells. Hepatology. (2016) 63:173–84. doi: 10.1002/hep.28251 PMC4688087 26403645

[87] Duarte TL Talbot NP Drakesmith H. NRF2 and hypoxia-inducible factors: key players in the redox control of systemic iron homeostasis. Antioxid Redox Signal. (2021) 35:433–52. doi: 10.1089/ars.2020.8148 32791852

[88] Wang Y Zhang L Zhou X. Activation of Nrf2 signaling protects hypoxia-induced HTR-8/SVneo cells against ferroptosis. J Obstet Gynaecol Res. (2021) 47:3797–806. doi: 10.1111/jog.15009 34525490

[89] Zhu H Jia Z Misra BR Zhang L Cao Z Yamamoto M. Nuclear factor E2-related factor 2-dependent myocardiac cytoprotection against oxidative and electrophilic stress. Cardiovasc Toxicol. (2008) 8:71–85. doi: 10.1007/s12012-008-9016-0 18463988

[90] Kerins MJ Ooi A. The roles of NRF2 in modulating cellular iron homeostasis. Antioxid Redox Signal. (2018) 29:1756–73. doi: 10.1089/ars.2017.7176 PMC6208163 28793787

[91] Fan Z Wirth AK Chen D Wruck CJ Rauh M Buchfelder M. Nrf2-Keap1 pathway promotes cell proliferation and diminishes ferroptosis. Oncogenesis. (2017) 6:e371. doi: 10.1038/oncsis.2017.65 28805788 PMC5608917

[92] Dai E Han L Liu J Xie Y Kroemer G Klionsky DJ. Autophagy-dependent ferroptosis drives tumor-associated macrophage polarization via release and uptake of oncogenic KRAS protein. Autophagy. (2020) 16:2069–83. doi: 10.1080/15548627.2020.1714209 PMC7595620 31920150

[93] Kang R Tang D. Autophagy and ferroptosis - what’s the connection? Curr Pathobiol Rep. (2017) 5:153–9. doi: 10.1007/s40139-017-0139-5 PMC5640172 29038744

[94] Liu J Yang M Kang R Klionsky DJ Tang D. Autophagic degradation of the circadian clock regulator promotes ferroptosis. Autophagy. (2019) 15:2033–5. doi: 10.1080/15548627.2019.1659623 PMC6844535 31441366

[95] Wang H Liu C Zhao Y Gao G. Mitochondria regulation in ferroptosis. Eur J Cell Biol. (2020) 99:151058. doi: 10.1016/j.ejcb.2019.151058 31810634

[96] Hou W Xie Y Song X Sun X Lotze MT Zeh HJ 3rd. Autophagy promotes ferroptosis by degradation of ferritin. Autophagy. (2016) 12:1425–8. doi: 10.1080/15548627.2016.1187366 PMC4968231 27245739

[97] Bai Y Meng L Han L Jia Y Zhao Y Gao H. Lipid storage and lipophagy regulates ferroptosis. Biochem Biophys Res Commun. (2019) 508:997–1003. doi: 10.1016/j.bbrc.2018.12.039 30545638

[98] Babayev E Seli E. Oocyte mitochondrial function and reproduction. Curr Opin Obstet Gynecol. (2015) 27:175–81. doi: 10.1097/GCO.0000000000000164 PMC4590773 25719756

[99] Fan H He J Bai Y He Q Zhang T Zhang J. Baicalin improves the functions of granulosa cells and the ovary in aged mice through the mTOR signaling pathway. J Ovarian Res. (2022) 15:34. doi: 10.1186/s13048-022-00965-7 35300716 PMC8932175

[100] Zhang J Liu Y Yao W Li Q Liu H Pan Z. Initiation of follicular atresia: gene networks during early atresia in pig ovaries. Reproduction. (2018) 156:23–33. doi: 10.1530/REP-18-0058 29743261

[101] Tsai-Turton M Luong BT Tan Y Luderer U. Cyclophosphamide-induced apoptosis in COV434 human granulosa cells involves oxidative stress and glutathione depletion. Toxicol Sci. (2007) 98:216–30. doi: 10.1093/toxsci/kfm087 17434952

[102] Zhang D Liu Y Zhang Z Lv P Liu Y Li J. Basonuclin 1 deficiency is a cause of primary ovarian insufficiency. Hum Mol Genet. (2018) 27:3787–800. doi: 10.1093/hmg/ddy261 30010909

[103] Wang F Liu Y Ni F Jin J Wu Y Huang Y. BNC1 deficiency-triggered ferroptosis through the NF2-YAP pathway induces primary ovarian insufficiency. Nat Commun. (2022) 13:5871. doi: 10.1038/s41467-022-33323-8 36198708 PMC9534854

[104] Jakimiuk AJ Weitsman SR Navab A Magoffin DA. Luteinizing hormone receptor, steroidogenesis acute regulatory protein, and steroidogenic enzyme messenger ribonucleic acids are overexpressed in thecal and granulosa cells from polycystic ovaries. J Clin Endocrinol Metab. (2001) 86:1318–23. doi: 10.1210/jcem.86.3.7318 11238527

[105] Yang H Xie Y Yang D Ren D. Oxidative stress-induced apoptosis in granulosa cells involves JNK, p53 and Puma. Oncotarget. (2017) 8:25310–22. doi: 10.18632/oncotarget.v8i15 PMC5421932 28445976

[106] Shen M Cao Y Jiang Y Wei Y Liu H. Melatonin protects mouse granulosa cells against oxidative damage by inhibiting FOXO1-mediated autophagy: Implication of an antioxidation-independent mechanism. Redox Biol. (2018) 18:138–57. doi: 10.1016/j.redox.2018.07.004 PMC6068202 30014903

[107] Ni Z Li Y Song D Ding J Mei S Sun S. Iron-overloaded follicular fluid increases the risk of endometriosis-related infertility by triggering granulosa cell ferroptosis and oocyte dysmaturity. Cell Death Dis. (2022) 13:579. doi: 10.1038/s41419-022-05037-8 35787614 PMC9253011

[108] Mancias JD Pontano Vaites L Nissim S Biancur DE Kim AJ Wang X. Ferritinophagy via NCOA4 is required for erythropoiesis and is regulated by iron dependent HERC2-mediated proteolysis. Elife. (2015) 4:e10308. doi: 10.7554/eLife.10308 26436293 PMC4592949

[109] Wang S Ji LY Li L Li JM. Oxidative stress, autophagy and pyroptosis in the neovascularization of oxygen−induced retinopathy in mice. Mol Med Rep. (2019) 19:927–34. doi: 10.3892/mmr.2018.9759 PMC6323229 30569132

[110] Moreno-Navarrete JM Lopez-Navarro E Candenas L Pinto F Ortega FJ Sabater-Masdeu M. Ferroportin mRNA is down-regulated in granulosa and cervical cells from infertile women. Fertil Steril. (2017) 107:236–42. doi: 10.1016/j.fertnstert.2016.10.008 27842994

[111] Zhang D Yi S Cai B Wang Z Chen M Zheng Z. Involvement of ferroptosis in the granulosa cells proliferation of PCOS through the circRHBG/miR-515/SLC7A11 axis. Ann Transl Med. (2021) 9:1348. doi: 10.21037/atm 34532485 PMC8422124

[112] Nemati A Alipanah-Moghadam R Molazadeh L Naghizadeh Baghi A. The effect of glutamine supplementation on oxidative stress and matrix metalloproteinase 2 and 9 after exhaustive exercise. Drug Des Devel Ther. (2019) 13:4215–23. doi: 10.2147/DDDT PMC6912001 31849453

[113] Cacciottola L Donnez J Dolmans MM. Can endometriosis-related oxidative stress pave the way for new treatment targets? Int J Mol Sci. (2021) 22(13):7138. doi: 10.3390/ijms22137138 34281188 PMC8267660

[114] Saeed N Hamzah IH Al-Gharrawi SAR. Polycystic ovary syndrome dependency on mtDNA mutation; copy Number and its association with insulin resistance. BMC Res Notes. (2019) 12:455. doi: 10.1186/s13104-019-4453-3 31340838 PMC6657173

[115] Zhang M Bener MB Jiang Z Wang T Esencan E Scott R. Mitofusin 2 plays a role in oocyte and follicle development, and is required to maintain ovarian follicular reserve during reproductive aging. Aging (Albany NY). (2019) 11:3919–38. doi: 10.18632/aging.v11i12 PMC6628992 31204316

[116] Wang CH Wei YH. Role of mitochondrial dysfunction and dysregulation of Ca(2+) homeostasis in the pathophysiology of insulin resistance and type 2 diabetes. J BioMed Sci. (2017) 24:70. doi: 10.1186/s12929-017-0375-3 28882140 PMC5588717

[117] Kim JA Wei Y Sowers JR. Role of mitochondrial dysfunction in insulin resistance. Circ Res. (2008) 102:401–14. doi: 10.1161/CIRCRESAHA.107.165472 PMC2963150 18309108

[118] Yang S Lian G. ROS and diseases: role in metabolism and energy supply. Mol Cell Biochem. (2020) 467:1–12. doi: 10.1007/s11010-019-03667-9 31813106 PMC7089381

[119] Zhang J Bao Y Zhou X Zheng L. Polycystic ovary syndrome and mitochondrial dysfunction. Reprod Biol Endocrinol. (2019) 17:67. doi: 10.1186/s12958-019-0509-4 31420039 PMC6698037

[120] Papalou O Victor VM Diamanti-Kandarakis E. Oxidative stress in polycystic ovary syndrome. Curr Pharm Des. (2016) 22:2709–22. doi: 10.2174/1381612822666160216151852 26881435

[121] Ding Y Xia BH Zhang CJ Zhuo GC. Mitochondrial tRNA(Leu(UUR)) C3275T, tRNA(Gln) T4363C and tRNA(Lys) A8343G mutations may be associated with PCOS and metabolic syndrome. Gene. (2018) 642:299–306. doi: 10.1016/j.gene.2017.11.049 29155328

[122] Zhao H Zhao Y Li T Li M Li J Li R. Metabolism alteration in follicular niche: The nexus among intermediary metabolism, mitochondrial function, and classic polycystic ovary syndrome. Free Radic Biol Med. (2015) 86:295–307. doi: 10.1016/j.freeradbiomed.2015.05.013 26057937

[123] Liu H Xie J Fan L Xia Y Peng X Zhou J. Cryptotanshinone Protects against PCOS-Induced Damage of Ovarian Tissue via Regulating Oxidative Stress, Mitochondrial Membrane Potential, Inflammation, and Apoptosis via Regulating Ferroptosis. Oxid Med Cell Longev. (2022) 2022:8011850. doi: 10.1155/2022/8011850 35419170 PMC9001078

[124] Boirie Y. Insulin regulation of mitochondrial proteins and oxidative phosphorylation in human muscle. Trends Endocrinol Metab. (2003) 14:393–4. doi: 10.1016/j.tem.2003.09.002 14580754

[125] Evans JL Maddux BA Goldfine ID. The molecular basis for oxidative stress-induced insulin resistance. Antioxid Redox Signal. (2005) 7:1040–52. doi: 10.1089/ars.2005.7.1040 15998259

[126] Babaei-Abraki S Karamali F Nasr-Esfahani MH. Monitoring the induction of ferroptosis following dissociation in human embryonic stem cells. J Biol Chem. (2022) 298:101855. doi: 10.1016/j.jbc.2022.101855 35337799 PMC9034286

[127] Zhang Y Hu M Jia W Liu G Zhang J Wang B. Hyperandrogenism and insulin resistance modulate gravid uterine and placental ferroptosis in PCOS-like rats. J Endocrinol. (2020) 246:247–63. doi: 10.1530/JOE-20-0155 32590339

[128] Beharier O Tyurin VA Goff JP Guerrero-Santoro J Kajiwara K Chu T. PLA2G6 guards placental trophoblasts against ferroptotic injury. Proc Natl Acad Sci USA. (2020) 117:27319–28. doi: 10.1073/pnas.2009201117 PMC7959495 33087576

[129] Rodriguez Paris V Wong XYD Solon-Biet SM Edwards MC Aflatounian A Gilchrist RB. The interplay between PCOS pathology and diet on gut microbiota in a mouse model. Gut Microbes. (2022) 14:2085961. doi: 10.1080/19490976.2022.2085961 35787106 PMC9450977

[130] Song X Long D. Nrf2 and ferroptosis: A new research direction for neurodegenerative diseases. Front Neurosci. (2020) 14:267doi: 10.3389/fnins.2020.00267 32372896 PMC7186402

[131] Zhu T Cui J Goodarzi MO. Polycystic ovary syndrome and risk of type 2 diabetes, coronary heart disease, and stroke. Diabetes. (2021) 70:627–37. doi: 10.2337/db20-0800 33158931

[132] Greenwood EA Huddleston HG. Insulin resistance in polycystic ovary syndrome: concept versus cutoff. Fertil Steril. (2019) 112:827–8. doi: 10.1016/j.fertnstert.2019.08.100 31731944

[133] Mahalingaiah S Diamanti-Kandarakis E. Targets to treat metabolic syndrome in polycystic ovary syndrome. Expert Opin Ther Targets. (2015) 19:1561–74. doi: 10.1517/14728222.2015.1101067 PMC4883581 26488852

[134] Yilmaz M Bukan N Ayvaz G Karakoc A Toruner F Cakir N. The effects of rosiglitazone and metformin on oxidative stress and homocysteine levels in lean patients with polycystic ovary syndrome. Hum Reprod. (2005) 20:3333–40. doi: 10.1093/humrep/dei258 16123091

[135] Teede HJ Misso ML Costello MF Dokras A Laven J Moran L. Recommendations from the international evidence-based guideline for the assessment and management of polycystic ovary syndrome. Hum Reprod. (2018) 33:1602–18. doi: 10.1093/humrep/dey256 PMC6112576 30052961

[136] Li J Cao F Yin HL Huang ZJ Lin ZT Mao N. Ferroptosis: past, present and future. Cell Death Dis. (2020) 11:88. doi: 10.1038/s41419-020-2298-2 32015325 PMC6997353

[137] Liu S Navarro G Mauvais-Jarvis F. Androgen excess produces systemic oxidative stress and predisposes to beta-cell failure in female mice. PloS One. (2010) 5:e11302. doi: 10.1371/journal.pone.0011302 20585581 PMC2892018

[138] Chappel S. The role of mitochondria from mature oocyte to viable blastocyst. Obstet Gynecol Int. (2013) 2013:183024. doi: 10.1155/2013/183024 23766762 PMC3671549

[139] Escobar-Morreale HF Luque-Ramirez M Alvarez-Blasco F Botella-Carretero JI Sancho J San Millan JL. Body iron stores are increased in overweight and obese women with polycystic ovary syndrome. Diabetes Care. (2005) 28:2042–4. doi: 10.2337/diacare.28.8.2042 16043756

[140] Escobar-Morreale HF Luque-Ramirez M. Role of androgen-mediated enhancement of erythropoiesis in the increased body iron stores of patients with polycystic ovary syndrome. Fertil Steril. (2011) 95:1730–5 e1. doi: 10.1016/j.fertnstert.2011.01.038 21300335

[141] Escobar-Morreale HF San Millan JL. Abdominal adiposity and the polycystic ovary syndrome. Trends Endocrinol Metab. (2007) 18:266–72. doi: 10.1016/j.tem.2007.07.003 17693095

[142] Wang H Wang X Zhu Y Chen F Sun Y Han X. Increased androgen levels in rats impair glucose-stimulated insulin secretion through disruption of pancreatic beta cell mitochondrial function. J Steroid Biochem Mol Biol. (2015) 154:254–66. doi: 10.1016/j.jsbmb.2015.09.003 26348137

[143] Luque-Ramirez M Alvarez-Blasco F Alpanes M Escobar-Morreale HF. Role of decreased circulating hepcidin concentrations in the iron excess of women with the polycystic ovary syndrome. J Clin Endocrinol Metab. (2011) 96:846–52. doi: 10.1210/jc.2010-2211 21209031

[144] Mou Y Wang J Wu J He D Zhang C Duan C. Ferroptosis, a new form of cell death: opportunities and challenges in cancer. J Hematol Oncol. (2019) 12:34. doi: 10.1186/s13045-019-0720-y 30925886 PMC6441206

[145] Hu M Zhang Y Ma S Li J Wang X Liang M. Suppression of uterine and placental ferroptosis by N-acetylcysteine in a rat model of polycystic ovary syndrome. Mol Hum Reprod. (2021) 27(12):gaab067. doi: 10.1093/molehr/gaab067 34850077

[146] Sun L Hu W Liu Q Hao Q Sun B Zhang Q. Metabonomics reveals plasma metabolic changes and inflammatory marker in polycystic ovary syndrome patients. J Proteome Res. (2012) 11:2937–46. doi: 10.1021/pr3000317 22428626

[147] Li S Chu Q Ma J Sun Y Tao T Huang R. Discovery of novel lipid profiles in PCOS: do insulin and androgen oppositely regulate bioactive lipid production? J Clin Endocrinol Metab. (2017) 102:810–21. doi: 10.1210/jc.2016-2692 PMC5477809 27886515

[148] Huang R Xue X Li S Wang Y Sun Y Liu W. Alterations of poly-unsaturated fatty acid metabolism in ovarian tissues of polycystic ovary syndrome rats. J Cell Mol Med. (2018) 22:3388–96. doi: 10.1111/jcmm.13614 PMC6010729 29602230

[149] Sak S Uyanikoglu H Incebiyik A Incebiyik H Hilali NG Sabuncu T. Associations of serum fetuin-A and oxidative stress parameters with polycystic ovary syndrome. Clin Exp Reprod Med. (2018) 45:116–21. doi: 10.5653/cerm.2018.45.3.116 PMC6125147 30202741

[150] Raizel R Leite JS Hypolito TM Coqueiro AY Newsholme P Cruzat VF. Determination of the anti-inflammatory and cytoprotective effects of l-glutamine and l-alanine, or dipeptide, supplementation in rats submitted to resistance exercise. Br J Nutr. (2016) 116:470–9. doi: 10.1017/S0007114516001999 27215379

[151] Ge MH Tian H Mao L Li DY Lin JQ Hu HS. Zinc attenuates ferroptosis and promotes functional recovery in contusion spinal cord injury by activating Nrf2/GPX4 defense pathway. CNS Neurosci Ther. (2021) 27:1023–40. doi: 10.1111/cns.13657 PMC8339532 33951302

[152] Uckan K Demir H Turan K Sarikaya E Demir C. Role of oxidative stress in obese and nonobese PCOS patients. Int J Clin Pract. (2022) 2022:4579831. doi: 10.1155/2022/4579831 35685525 PMC9159123

[153] Malin SK Kirwan JP Sia CL Gonzalez F. Glucose-stimulated oxidative stress in mononuclear cells is related to pancreatic beta-cell dysfunction in polycystic ovary syndrome. J Clin Endocrinol Metab. (2014) 99:322–9. doi: 10.1210/jc.2013-3177 PMC3879676 24203060

[154] Palacio JR Iborra A Ulcova-Gallova Z Badia R Martinez P. The presence of antibodies to oxidative modified proteins in serum from polycystic ovary syndrome patients. Clin Exp Immunol. (2006) 144:217–22. doi: 10.1111/j.1365-2249.2006.03061.x PMC1809652 16634794

[155] Chen PH Tseng WH Chi JT. The intersection of DNA damage response and ferroptosis-A rationale for combination therapeutics. Biol (Basel). (2020) 9(8):187. doi: 10.3390/biology9080187 PMC7464484 32718025

[156] Ryu Y Kim SW Kim YY Ku SY. Animal models for human polycystic ovary syndrome (PCOS) focused on the use of indirect hormonal perturbations: A review of the literature. Int J Mol Sci. (2019) 20(11):2720. doi: 10.3390/ijms20112720 31163591 PMC6600358

[157] Choi HD Kim JH Chang MJ Kyu-Youn Y Shin WG. Effects of astaxanthin on oxidative stress in overweight and obese adults. Phytother Res. (2011) 25:1813–8. doi: 10.1002/ptr.3494 21480416

[158] Azziz R Carmina E Chen Z Dunaif A Laven JS Legro RS. Polycystic ovary syndrome. Nat Rev Dis Primers. (2016) 2:16057. doi: 10.1038/nrdp.2016.57 27510637

[159] Joham AE Piltonen T Lujan ME Kiconco S Tay CT. Challenges in diagnosis and understanding of natural history of polycystic ovary syndrome. Clin Endocrinol (Oxf). (2022) 97:165–73. doi: 10.1111/cen.14757 PMC9541175 35593530

[160] Jones DP. Redefining oxidative stress. Antioxid Redox Signal. (2006) 8:1865–79. doi: 10.1089/ars.2006.8.1865 16987039

[161] Sulaiman MA Al-Farsi YM Al-Khaduri MM Saleh J Waly MI. Polycystic ovarian syndrome is linked to increased oxidative stress in Omani women. Int J Womens Health. (2018) 10:763–71. doi: 10.2147/IJWH PMC6276615 30568513

[162] Hoeger KM Dokras A Piltonen T. Update on PCOS: consequences, challenges, and guiding treatment. J Clin Endocrinol Metab. (2021) 106:e1071–e83. doi: 10.1210/clinem/dgaa839 33211867

[163] Deswal R Narwal V Dang A Pundir CS. The prevalence of polycystic ovary syndrome: A brief systematic review. J Hum Reprod Sci. (2020) 13:261–71. doi: 10.4103/jhrs.JHRS_95_18 PMC7879843 33627974

[164] Kakoly NS Earnest A Teede HJ Moran LJ Joham AE. The impact of obesity on the incidence of type 2 diabetes among women with polycystic ovary syndrome. Diabetes Care. (2019) 42:560–7. doi: 10.2337/dc18-1738 30705063

[165] Zheng R Lin C Mao Y Jin F. miR-761-hepcidin/Gpx4 pathway contribute to unexplained liver dysfunction in polycystic ovary syndrome by regulating liver iron overload and ferroptosis. Gynecol Endocrinol. (2023) 39:2166483. doi: 10.1080/09513590.2023.2166483 36657482

[166] Tang H Jiang X Hua Y Li H Zhu C Hao X. NEDD4L facilitates granulosa cell ferroptosis by promoting GPX4 ubiquitination and degradation. Endocr Connect. (2023) 12(4):e220459. doi: 10.1530/EC-22-0459 36662677 PMC10083675

[167] Jiang Y Yang J Du K Luo K Yuan X Hua F. 1,25-Dihydroxyvitamin D3 alleviates hyperandrogen-induced ferroptosis in KGN cells. Hormones (Athens). (2023) 22:273–80. doi: 10.1007/s42000-023-00439-5 PMC10209298 36884209

[168] Zhang J Ding N Xin W Yang X Wang F. Quantitative proteomics reveals that a prognostic signature of the endometrium of the polycystic ovary syndrome women based on ferroptosis proteins. Front Endocrinol (Lausanne). (2022) 13:871945.doi: 10.3389/fendo.2022.871945 35909514 PMC9330063

[169] Lin S Jin X Gu H Bi F. Relationships of ferroptosis-related genes with the pathogenesis in polycystic ovary syndrome. Front Med (Lausanne). (2023) 10:1120693.doi: 10.3389/fmed.2023.1120693 36873892 PMC9981782

[170] Tan W Dai F Yang D Deng Z Gu R Zhao X. MiR-93-5p promotes granulosa cell apoptosis and ferroptosis by the NF-kB signaling pathway in polycystic ovary syndrome. Front Immunol. (2022) 13:967151.doi: 10.3389/fimmu.2022.967151 36341347 PMC9626535

[171] Zhang P Pan Y Wu S He Y Wang J Chen L. n-3 PUFA promotes ferroptosis in PCOS GCs by inhibiting YAP1 through activation of the hippo pathway. Nutrients. (2023) 15(8):1927. doi: 10.3390/nu15081927 37111146 PMC10145554

[172] Zhang L Wang F Li D Yan Y Wang H. Transferrin receptor-mediated reactive oxygen species promotes ferroptosis of KGN cells via regulating NADPH oxidase 1/PTEN induced kinase 1/acyl-CoA synthetase long chain family member 4 signaling. Bioengineered. (2021) 12:4983–94. doi: 10.1080/21655979.2021.1956403 PMC8806504 34369274

[173] Peng Q Chen X Liang X Ouyang J Wang Q Ren S. Metformin improves polycystic ovary syndrome in mice by inhibiting ovarian ferroptosis. Front Endocrinol (Lausanne). (2023) 14:1070264.doi: 10.3389/fendo.2023.1070264 36755918 PMC9900736

